# Regulation of Mitochondrial Function by the Actin Cytoskeleton

**DOI:** 10.3389/fcell.2021.795838

**Published:** 2021-12-21

**Authors:** María Illescas, Ana Peñas, Joaquín Arenas, Miguel A. Martín, Cristina Ugalde

**Affiliations:** ^1^ Instituto de Investigación Hospital 12 de Octubre, Madrid, Spain; ^2^ Centro de Investigación Biomédica en Red de Enfermedades Raras (CIBERER), Madrid, Spain

**Keywords:** mitochondria, actin cytoskeleton, OXPHOS system, gelsolin, mitochondrial disease

## Abstract

The regulatory role of actin cytoskeleton on mitochondrial function is a growing research field, but the underlying molecular mechanisms remain poorly understood. Specific actin-binding proteins (ABPs), such as Gelsolin, have also been shown to participate in the pathophysiology of mitochondrial OXPHOS disorders through yet to be defined mechanisms. In this mini-review, we will summarize the experimental evidence supporting the fundamental roles of actin cytoskeleton and ABPs on mitochondrial trafficking, dynamics, biogenesis, metabolism and apoptosis, with a particular focus on Gelsolin involvement in mitochondrial disorders. The functional interplay between the actin cytoskeleton, ABPs and mitochondrial membranes for the regulation of cellular homeostasis thus emerges as a new exciting field for future research and therapeutic approaches.

## Introduction

As a major component of the cellular structural network, relevant biological processes like cell division, migration, intracellular transport and organelle organization extensively rely on the dynamics and organization of the actin cytoskeleton. Actin filaments (F-actin) are formed by the polymerization of globular actin monomers (G-actin) in a neat disposition that allows filaments to be polarized. Their remodeling is controlled by a repertoire of actin-binding proteins (ABPs), expressed in a tissue-dependent manner depending on where actin executes cell-specific functions (Lappalainen, 2016; Merino et al., 2020). These proteins regulate a wide spectrum of cellular processes, and are classified regarding their specific action mechanisms: maintenance of the G-actin monomers pool; G-actin nucleation and polymerization of actin filaments and branches; and filaments severing and depolymerization, mainly driven by cofilin and the gelsolin protein superfamily (Silacci et al., 2004).

## Actin Cytoskeleton Involvement on Mitochondrial Function

Mitochondria are present in eukaryotic cells and possess a characteristic architecture. The outer mitochondrial membrane (OMM) surrounds the inner mitochondrial membrane (IMM) creating two separate compartments: the internal matrix and intermembrane space (IMS). Mitochondria are fundamental for reactive oxygen species (ROS) production, calcium homeostasis, heat production, cell proliferation or apoptosis (Brookes et al., 2004), and are the main site to important metabolic reactions including the citric acid cycle, amino acids interconversion or β-oxidation of fatty acids (Nunnari and Suomalainen, 2012), and ATP synthesis through the oxidative phosphorylation (OXPHOS) system (Reid et al., 1966).

Actin is mainly located at the cell membrane, but also at specific mitochondrial subpopulations (Venit et al., 2021). Interactions between mitochondria and the actin cytoskeleton link the essential functions of this organelle to a plethora of cellular physiological processes. Actin filaments primarily modulate mitochondrial dynamics (Moore et al., 2016; Tilokani et al., 2018), trafficking and autophagy (Kast and Dominguez, 2017), but also mitochondrial biogenesis and metabolism (Fernie et al., 2020). The purpose of this review is to highlight the often overlooked regulatory roles of actin cytoskeleton and ABPs on mitochondrial function.

### Actin Cytoskeleton on Mitochondrial Dynamics

Mitochondrial function directly depends on its correct morphology and distribution (Scott and Youle, 2010; Sheng, 2014; Chan, 2020), controlled by the balance between fission (division into two or more independent organelles), fusion (formation of a single structure) and mitophagy (clearance of damaged organelles) (Ni et al., 2015). Due to the importance of this system for the maintenance of the cellular metabolic state in mammals (Youle and Van Der Bliek, 2012), the fission and fusion forces require a high degree of regulation by specific molecules along with the actin cytoskeleton. In fact, the dynamic cycling of actin between mitochondrial subpopulations regulates mitochondrial motility and the fission–fusion balance within mitochondrial networks (Moore et al., 2016).

**FIGURE 1:**
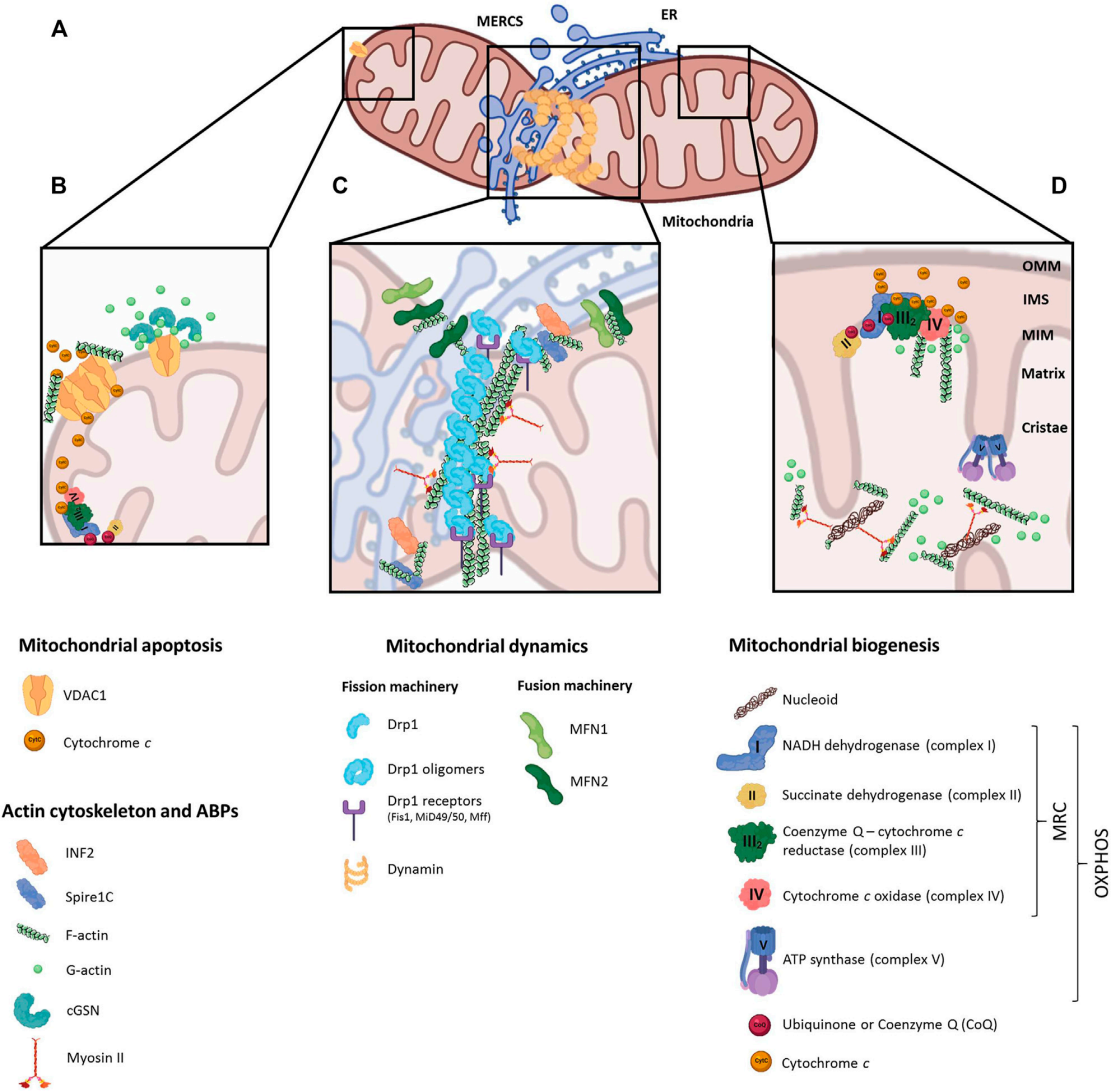
Regulatory roles of actin cytoskeleton on mitochondrial function. **(A)** Schematic representation of a mitochondrion interacting with the endoplasmic reticulum (ER) at mitochondrial-endoplasmic reticulum contact sites (MERCS), where dynamin is recruited and fission events take place. **(B)** Under anti-apoptotic stimuli, actin-binding proteins interact with the voltage-dependent anion channel 1 (VDAC1) in the outer mitochondrial membrane (OMM), preventing actin filaments oligomerization and apoptosis; conversely, actin polymerization favors VDAC oligomerization and induces the apoptotic pathway. **(C)** Actin cytoskeleton is represented at MERCS, together with ABPs (INF2, Spire1C) and OMM proteins involved in mitochondrial dynamics (MFN1/2). Actin and myosin II localization at MERCSs initiates Drp1 recruitment, which binding to Drp1 receptors (Fis1, MUD49/50, MFF) leads to mitochondrial fission. **(D)** Actin interacts with mitochondrial respiratory chain (MRC) complexes III and IV in the matrix, controlling the association and dissociation of cytochrome *c*; and together with myosinII, actin regulates the distribution of mitochondrial nucleoids.

The main role of actin in mitochondrial dynamics is closely linked to the formation of mitochondria-endoplasmic reticulum contacts (MERCs), known as ERMES (ER-mitochondria encounter structure) in yeast (Kornmann and Walter, 2010) (Figure 1A). MERCs are involved in many biological processes like calcium signaling, autophagy, mtDNA replication and phospholipid trafficking (Bononi et al., 2012; Lewis et al., 2016; Xu et al., 2020), besides mitochondrial fission and fusion (Guo et al., 2018; Abrisch et al., 2020). The fusion of adjacent OMMs is orchestrated by mitofusins 1 and 2 (MFN1/2), outer mitochondrial membrane GTPases that form homo- and heterodimers (Friedman et al., 2011). MFN2 is known to tether MERCs, regulating mitochondrial calcium uptake from ER (Han et al., 2021). MERCs are formed before the recruitment of the fission machinery, defining the position of mitochondrial fission sites in a process called ERMD (ER-associated mitochondrial division) (Friedman et al., 2011). The ER-anchored formin INF2 binds to the OMM-located actin nucleator Spire1C, leading to the polymerization of F-actin at MERCs (Korobova et al., 2013; Manor et al., 2015). This leads to the pre-constriction of the OMM driven by the joint action of the ER, actin and non-muscular myosin II. Actin and myosin II ultimately recruit Drp1 (Dynamin Related Protein 1) from the cytosol to the OMM (De Vos et al., 2005), where it oligomerizes (Ji et al., 2015) and interacts with mitochondrial receptors (Mff, MiD49/51 or Fis1), shaping a ring that further constricts and splits the mitochondrion by GTP hydrolysis (Francy et al., 2015). Furthermore, INF2-mediated actin polymerization stimulates the mitochondrial calcium spike, enhancing the metabolic flux by the OXPHOS system and prompting IMM constriction at later stages (Chakrabarti et al., 2018). Interestingly, human cellular models depleted of ABPs, such as spire1C, myosin II or cofilin, showed abnormal mitochondrial motility and morphology, and altered Drp1 recruitment to the OMM (Korobova et al., 2013, 2014; Manor et al., 2015; Pagliuso et al., 2016; Rehklau et al., 2017), pointing to their dual role in OMM pre-constriction and recruitment of fission proteins to the MERCs **(** Figure 1C).

### Actin Cytoskeleton on Mitochondrial Trafficking

Long-range mitochondrial transport has been primarily studied in neurons (Hirokawa and Takemura, 2005; Schwarz, 2013). This process is mediated by the coupling of mitochondria to microtubule motor proteins (kinesins and dyneins), forming a motor complex together with the OMM GTPase Miro and the adaptor protein Milton/TRAK that mediates mitochondrial transport (van Spronsen et al., 2013; Kruppa and Buss, 2021). Mitochondrial trafficking is closely regulated by intracellular calcium levels, whose increase inhibits both kinesin-dependent anterograde and dynein-dependent retrograde movements (Rintoul et al., 2003); by the PINK1/Parkin pathway, which regulates Miro turnover (Wang et al., 2011; Birsa et al., 2014); or by the nerve growth factor (NGF), responsible for mitochondrial accumulation at the axon region closest to the bead in a manner that involves PI3 kinase and actin (Verburg and Hollenbeck, 2008). The Miro proteins are also key adaptors for the recruitment and stabilization of the ABP myosin19 (Myo19) to the mitochondria (Shneyer et al., 2016; López-Doménech et al., 2018). In human cells, Myo19 has been proposed to regulate the equal segregation of mitochondria to daughter cells during mitosis (Rohn et al., 2014). In yeast, after mitochondrial fission and prior to cell division, mitochondrial populations suffer a poleward movement to both sides of the division plane, exhibiting similar patterns as chromosomes, to contribute to an equitable mitochondrial inheritance to both daughter cells under actin-dependent mechanisms (Boldogh and Pon, 2006; Scott and Youle, 2010). Based on the similarities with cytokinesis, this process was defined as “mitokinesis” (Hatch et al., 2014).

### Actin Cytoskeleton on mtDNA Expression and Maintenance

The mammalian mitochondrial DNA genome (mtDNA) contains 37 genes organized in compact DNA:protein complexes called nucleoids (Clayton, 1991; Spelbrink, 2010), whose expression requires a high degree of coordination with the nuclear genome (Rampelt and Pfanner, 2016). In yeast, the ERMES complex regulates the stability and organization of mtDNA in nucleoids in an actin-dependent manner (Boldogh and Pon, 2006); in mammals, MERCs are spatially-linked to mitochondrial nucleoids, regulating their distribution, division and active transportation by the microtubules (Lewis et al., 2016). Although the presence of actin inside mitochondria and its functional connection with mtDNA was debated for decades (Venit et al., 2021), recent super-resolution microscopy-based studies probed the presence of β-actin-containing structures inside the mitochondrial matrix (Dadsena et al., 2021). Moreover, human cells lacking β-actin showed higher sensitivity to stress caused by loss of mitochondrial membrane potential (ΔΨ) plus perturbed mtDNA mass and nucleoid organization (Xie et al., 2018), suggesting a regulatory role in mtDNA transcription and quality control. Besides actin, myosin II is also associated with purified mitochondrial nucleoids, and its silencing produces mtDNA abnormalities (Reyes et al., 2011). These evidences support the role of actin and ABPs in mitochondrial nucleoid segregation and mtDNA transcription and maintenance, likely through formation of a “mitoskeleton” network supporting mtDNA inheritance **(** Figure 1D **)**.

### Actin Cytoskeleton on Mitochondrial Metabolism

Actin filaments are indispensable for the full activation of metabolic pathways that subsequently regulate mitochondrial function. For instance, activation of glycolytic enzymes such as aldolase or glyceraldehyde phosphate dehydrogenase, may occur through their direct binding to F-actin (Arnold and Pette, 1968). Aldolase is trapped by the actin cytoskeleton, whose release under PI3K activation increases aldolase activity, thus enhancing glycolysis (Hu et al., 2016). F-actin bundles may also sequester TRIM21 (Tripartite Motif-containing Protein 21), reducing the access of this E3 ubiquitin ligase to its substrates, such as the rate-limiting metabolic enzyme phosphofructokinase (PFK), thus maintaining high glycolytic rates (Park et al., 2020).

Interestingly, in brain mitochondria actin regulates the retention of cytochrome *c* between respiratory chain complexes III and IV by its direct association with both complexes, and inhibition of actin polymerization with cytochalasin *b* enhanced mitochondrial respiration through increased complex IV activity (Takahashi et al., 2018) **(** Figure 1D **)**.

### Actin Cytoskeleton on Apoptosis

Mitochondria represent a major component in the cellular apoptotic machinery (Dadsena et al., 2021), influencing relevant processes like development, cell turnover or immune responses (Man, 2016). During the intrinsic pathway, increased OMM permeabilization and cristae disorganization initiate the release of pro-apoptotic factors (such as cytochrome *c* or AIF) from the IMS into the cytosol, prompting the formation of the apoptosome protein complex that activates caspases and subsequent chromatin fragmentation (Portt et al., 2011). Actin itself is a substrate of caspase-mediated cleavage (Kayalar et al., 1996), leading to a 15-kDa fragment that can be N-myristoylated and targeted to mitochondria (Utsumi et al., 2003). This interaction modulates ROS production through the regulation of OMM permeabilization by opening-closing membrane channels. In yeast, monomeric actin interacts with the voltage-dependent anion channel (VDAC), thus impacting on apoptosis modulation via interfering with the exchange of metabolites and energy between mitochondria and the cytosol (Xu et al., 2001; Roman et al., 2006). Disruption of actin dynamics causes a dramatic loss of mitochondrial ΔΨ, increased ROS and cell death (Gourlay and Ayscough, 2005). Studies in human cell lines treated with actin-disrupting drugs, affecting both actin stabilization and depolymerization, also reinforced the requirement of actin remodeling for the induction of the intrinsic apoptosis pathway (Odaka et al., 2000; Yamazaki et al., 2000) (Figure 1B).

Many ABPs are actively involved in apoptosis regulation in both yeast and mammals (Franklin-Tong and Gourlay, 2008). One well-studied ABP participating in this process is cofilin. During apoptosis induction in mammalian cells cofilin loses its actin-binding affinity, being translocated to the mitochondria prior to the permeability transition pore (mPTP) opening that promotes cytochrome *c* release and apoptosis progression (Chua et al., 2003; Roh et al., 2013). This process could be mediated by the interaction of cofilin with Drp1 (Hu et al., 2020), although the binding of cofilin to G-actin seems enough to induce its mitochondrial translocation (Rehklau et al., 2012). Under oxidative stress, oxidized cofilin is also translocated to the mitochondria, promoting mitochondrial fission and triggering the release of cytochrome *c* leading to apoptosis (Klamt et al., 2009; Lapeña-Luzón et al., 2021). Other relevant ABPs of the Gelsolin protein superfamily, like gelsolin itself and villin, modulate apoptosis induction in the gastrointestinal epithelium (Wang et al., 2012; Roy et al., 2018). Villin is a tissue-specific actin-modifying protein (Khurana and George, 2008), which together with gelsolin are targeted to the mitochondria on early steps of the apoptotic pathway (Roy et al., 2018). Their ability to associate with both actin and mitochondria suggest their role in cell survival through the preservation of actin cytoskeleton dynamics in mitochondrial regions controlling the trafficking of anti- and pro-apoptotic signals.

Given the relevance of gelsolin in several aspects of mitochondrial pathophysiology, we will henceforth focus the review on this particular ABP.

## Gelsolin

Gelsolin (GSN) is an abundant ABP that participates in actin-remodeling either by sequestering G-actin or by severing, capping, and nucleating F-actin (Yin and Stossel, 1979; Sun et al., 1999; Feng et al., 2001). This is mediated by calcium concentration, phosphatidylinositol-4,5-bisphosphate (PIP_2_) and pH (Hu et al., 2016). In human, alternative splicing of *GSN* mRNA leads to two main isoforms with differentiated functions (Yin et al., 1984): the plasma (pGSN) and cytoplasmic (cGSN) isoforms (UniProtKB reference P06396). Structurally, both isoforms are composed by six gelsolin domains (G1-G6) (Kwiatkowski et al., 1986) divided in two homologous structures: the N-terminal fragment (G1-G3) is involved in actin severing, and the C-terminal fragment (G4-G6) coordinates calcium binding (Choe et al., 2002). Domains G3 and G4 are separated by a linker sequence of 70 amino acids that is prone to cleavage by caspase-3 (Kothakota et al., 1997; Kamada et al., 1998)*.*

### Plasma GSN

Secreted pGSN (86 kDa) differs from intracellular cGSN (81 kDa) in its N-terminal sequence, spanning a 51-amino acid secretory peptide, and in the presence of a disulphide bond between cysteine residues 188–201 that enhances its stability in the extracellular media (Kwiatkowski et al., 1988; Wen et al., 1996). pGSN modulates bacterial immune response, acting as a buffering agent in inflammation (Bucki et al., 2008b; Cheng et al., 2017), and it is a part of the extracellular actin scavenger system (EASS) responsible for rapid severing and clearance of actin filaments released from dead cells into the bloodstream (Lind et al., 1986; Lee and Galbraith, 1992). In conditions of massive cell death, substantial actin release overwhelms the EASS, resulting in a decline of circulating pGSN levels. Consequently, pGSN has been proposed as a biomarker for multiple diseases (Li et al., 2012), ranging from cardiovascular pathologies (Khatri et al., 2014; Piktel et al., 2018; Feldt et al., 2019) to major trauma, diabetes, Alzheimer’s disease, rheumatoid arthritis, sepsis, liver failure, or cancer, to the point that pGSN has been proposed as a general biomarker of health prognosis (Peddada et al., 2012).

### Cytosolic GSN

Cytosolic GSN (cGSN) is ubiquitously expressed and, besides its main role in actin filament remodeling, it participates in regulatory signaling pathways that require a continuous rearrangement of the actin cytoskeleton, such as the phospholipase C (PLC) or phosphoinositide 3-kinase (PI3K) cascades (Singh et al., 1996; Sun et al., 1997); the epidermal growth factor receptor (EGFR) pathway (Chen et al., 1996; Azuma et al., 1998; De Corte et al., 2002); phagocytosis mediated by the Fc-receptor or integrins (Serrander et al., 2000; Witke et al., 2001; Arora et al., 2004); and also as a transcriptional coactivator of the thyroid (TR) and androgen (AR) receptors (Nishimura et al., 2003; Kim et al., 2007), and of the hypoxia inducible factor (HIF-1) to favor hypoxia-regulated genes expression, *GSN* itself among them (Greijer et al., 2005; Li Q. et al., 2009). Finally, cGSN also interacts with p53, inhibiting its nucleus translocation (An et al., 2011).

Besides, cGSN is associated to membrane regions of the cell rich in actin filaments such as the ER, vesicles or mitochondrial membranes (Cooper et al., 1988; Hartwig et al., 1989). cGSN co-purifies with isolated mitochondria, where it interacts with the major OMM channel protein VDAC to promote cell survival responses (Figure 1B) (Koya et al., 2000; Kusano et al., 2000; García-Bartolomé et al., 2017). In fact, cGSN is as a dual regulator of apoptotic cell death due to its cleavage by caspase-3 in two independent fragments (Kothakota et al., 1997). The C-terminal fragment, of 41 kDa, as well as full-length cGSN, may act as anti-apoptotic factors through VDAC blockage, thus avoiding Cyt c release into the cytosol (Koya et al., 2000; Kusano et al., 2000). Both can also bind to actin and DNaseI, forming a ternary complex that prevents the nuclear translocation of DNaseI. In contrast, the pro-apoptotic N-terminal fragment, of 39 kDa, may severe actin filaments in a calcium-independent manner (Kothakota et al., 1997; Geng et al., 1998; Kamada et al., 1998). It competes with actin for DNaseI binding, releasing it from the GSN:actin:DNaseI ternary complex and promoting its nuclear translocation, ultimately leading to nuclear DNA degradation and apoptosis (Chhabra et al., 2005; Li Q. et al., 2009). Furthermore, cGSN overexpression may inhibit the apoptotic pathway by sequestering and inactivating caspase3 in a GSN:PIP2:caspase3 complex (Ohtsu et al., 1997), and by precluding nuclear translocation of p53 (An et al., 2011). This protective role of cGSN was also demonstrated in mouse models of Alzheimer´s disease, where cGSN overexpression prevented the cytotoxic effect induced by accumulation of the amyloid beta (Aβ) peptide on mitochondrial function and cell death (Qiao et al., 2005; Antequera et al., 2009). High levels of cGSN were also reported in experimental models mainly exhibiting oxidative stress, such as upon hydrogen peroxide treatment, (Chauhan et al., 2008; Ji et al., 2010), as a consequence of intracellular calcium alterations (Bucki et al., 2008a), under HIF-1-modulated hypoxia (Nishimura et al., 2003), and in pathophysiological alterations like ageing and senescence (Ahn, 2003), Down syndrome (Ji et al., 2009), and heart failure (Li G. H. et al., 2009; Patel et al., 2018).

### GSN and Mitochondrial Disease

The relative abundance of cGSN also increases in primary fibroblasts from patients and cellular models of OXPHOS system deficiency (Marín-Buera et al., 2015; García-Bartolomé et al., 2017; García-Bartolomé et al., 2020), suggesting its protective role through the regulation of cell survival responses. In these models, there was a reverse correlation between increased cGSN and decreased pGSN levels, resulting in a significantly high cGSN:pGSN protein ratio as a novel hallmark of OXPHOS dysfunction (García-Bartolomé et al., 2020). Interestingly, pGSN levels significantly decreased in OXPHOS-deficient patients, which reinforced the diagnosis accuracy for these disorders of the formerly reported biomarkers GDF-15 and FGF-21 (Peñas et al., 2021). These data suggest a tightly regulated coordination of both GSN isoforms, whose relevance in mitochondrial pathophysiology remains unknown.

## Conclusions and Perspectives

We have emphasized the importance of the actin cytoskeleton-mediated regulation on several aspects of mitochondrial (dys)function, and detailed the so-far known role of one of the most abundant ABPs, Gelsolin, in these processes. It remains unknown whether the apparently protective role of GSN, like other ABPs, directly impacts on mitochondrial function or whether it indirectly functions through regulation of actin cytoskeleton dynamics. Either way, the functional interplay between GSN isoforms in health and disease, as well as that between the actin cytoskeleton, ABPs and mitochondrial membranes for the regulation of cellular homeostasis and metabolism, open new exciting possibilities for future research.
